# Flow diverter implantation for CTA-negative giant vertebral artery dissection aneurysm: a case report

**DOI:** 10.3389/fradi.2025.1625207

**Published:** 2025-12-15

**Authors:** Jun-Ting Li, Jian-Min Liu, Kai-Jun Zhao

**Affiliations:** 1 Department of Neurosurgery, Shanghai East Hospital, School of Medicine, Tongji University, Shanghai, China; 2 Neurovascular Center, Changhai Hospital, Naval Medical University, Shanghai, China

**Keywords:** vertebral artery, hypoperfusion, VADA, CTA, flow diverter

## Abstract

**Objective:**

To evaluate the efficacy of flow diverter implantation for treating CTA-negative giant vertebral artery dissection aneurysm (VADA) and to address the challenges in lesion characterization using MRI.

**Methods:**

A 66-year-old male patient presented with a 3-month history of left facial numbness and dysarthria. Initial MRI-T1 revealed a mixed signal intensity lesion in the CPA region. However, both CTA and digital subtraction angiography (DSA) failed to identify any significant vascular abnormalities. Subsequently, CT-perfusion and dynamic contrast-enhanced computed tomography (DCE-CT) were performed to further characterize the lesion.

**Results:**

DCE-CT revealed a giant VADA, which was significantly larger than the lesion initially detected by MRI and was identified as the cause of hypoperfusion in the posterior circulation. Based on these findings, a flow diverter implantation procedure was performed successfully without complications. Angiographic follow-up at 8 months demonstrated no recurrence of the lesion. At the 14-month clinical follow-up, the patient exhibited complete resolution of symptoms, with a mRS score of 0, indicating an excellent functional outcome.

**Conclusion:**

Flow diverter implantation may be an effective treatment for CTA-negative giant VADAs. The limitations of MRI in accurately characterizing lesion size underscore the necessity of advanced imaging techniques, such as DCE-CT, for precise device selection and deployment.

## Introduction

**Figure 1:**
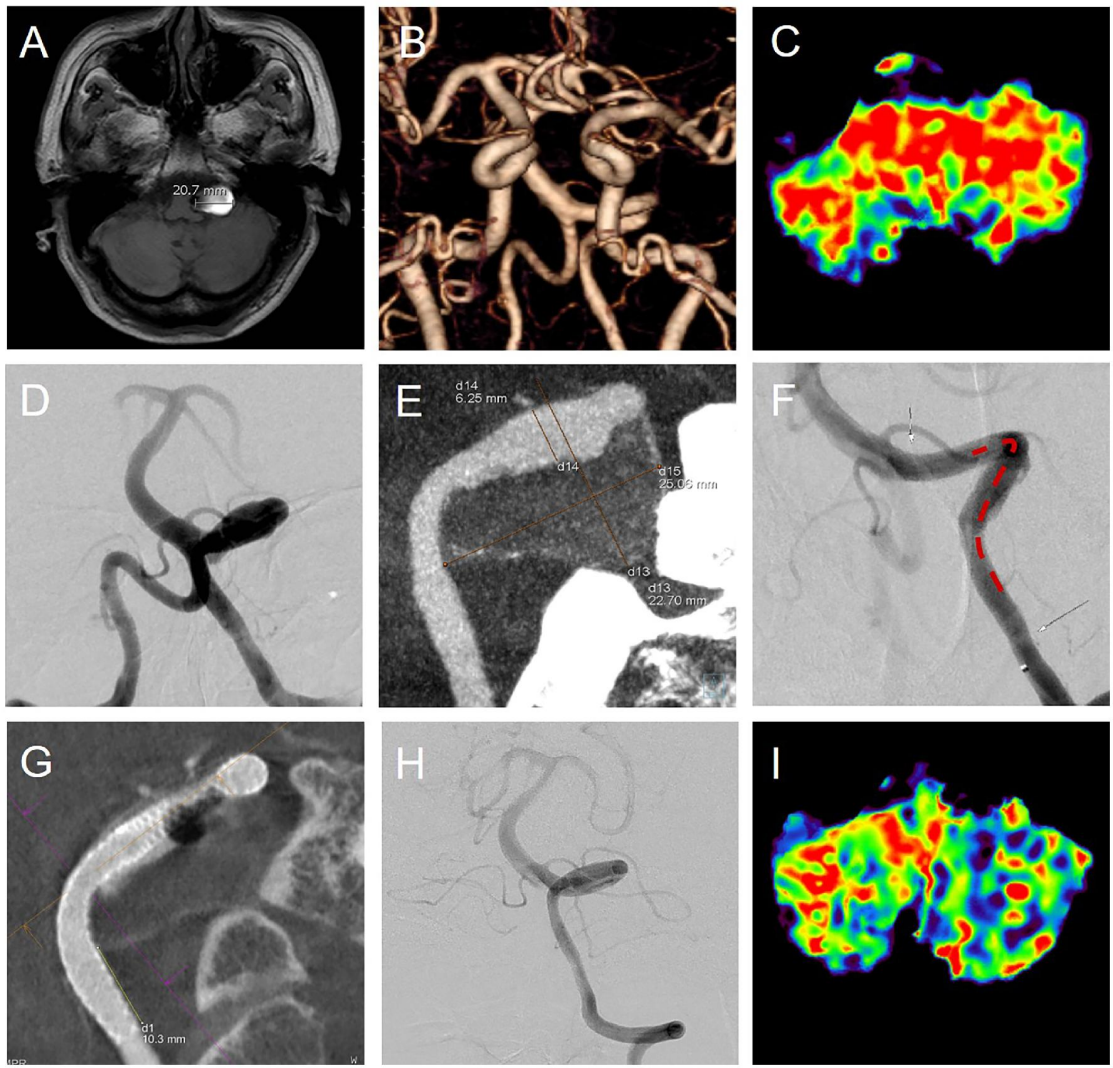
Preoperative assessment, treatment, and 8-month follow-up of a CTA-negative giant VADA. **(A)** Shows a mixed signal in the CPA region (max diameter: 20.7 mm). CTA **(B)** reveals normal V4 segment diameter, but **(C)** shows posterior circulation hypoperfusion. DSA **(D)** identifies left V4 tortuosity without abnormal dilation. DCE-CT **(E)** detects a giant left VADA (25.06 mm × 22.70 mm). A flow diverter (Surpass Evolve, 5 mm/40 mm) was implanted, extending 10 mm beyond the lesion margins (**F**; white arrows mark stent ends, red dashed line indicates lesion length). **(G)** confirms the stent extends 10.3 mm beyond the VADA edge. After 8 months **(H)**, facial numbness and dysarthria resolved, and posterior circulation perfusion normalized **(I)**.

Vertebral artery dissection (VAD) and its associated aneurysm (VADA) are significant causes of posterior circulation ischemia, particularly in young and middle-aged adults, and can also affect the elderly and children (1–3). The pathophysiology of VAD is characterized by dynamic vascular wall tearing, leading to potential underestimation of lesion extent and complex morphological changes on imaging (4). While initial magnetic resonance imaging (MRI, Figure 1A) is highly sensitive for detecting dissection aneurysms, it may not fully capture the true morphology of the lesion, especially in tortuous vessels. Computed tomography angiography (CTA, Figure 1B), though widely used, may miss some dissections or aneurysms (1). Advanced imaging techniques like dynamic contrast-enhanced computed tomography (DCE-CT, Figures 1E,G) offer higher accuracy in assessing lesion extent and guiding treatment (5, 6).

## Materials and methods

### Case presentation

A 66-year-old male patient presented with left facial numbness and dysarthria 3 months ago. Physical examination revealed clear consciousness, normal bilateral pupils, symmetric forehead wrinkles, no mouth corner drooping, and normal muscle strength and tone in all limbs. Babinski sign was positive on the right side. Brain MRI showed a mixed signal in the cerebellopontine angle (CPA) region. CTA revealed no significant vascular abnormalities. DSA demonstrated tortuosity near the confluence of the vertebral and basilar arteries in the left V4 segment without obvious dilation (Figure 1D). Given the ischemic hypoperfusion in the posterior circulation (Figure 1C), DCE-CT was performed, revealing a giant VADA in the left V4 segment, measuring 25.06 mm × 22.70 mm (Figure 1E), exceeding the maximum diameter shown on MRI-T1 (Figure 1A).

### Diagnosis

Giant VADA in the left V4 segment.

### Treatment and follow-up

Preoperatively, the patient was administered 300 mg of aspirin and 75 mg of clopidogrel for at least three days, then received systemic intravenous heparin, with the goal of an activated clotting time between 250 and 300 s under general anesthesia. A Surpass Evolve flow diverter (5 mm/40 mm, Stryker, Neurovascular, Fremont, CA) was implanted based on the extent of the left VADA shown on DCE-CT (Figures 1E,F). The stent was placed to fully cover the VADA, extending 10 mm beyond the proximal and distal margins of the aneurysm (Figures 1F–G).

During the perioperative period (Figure 1F) and at an 8-month angiographic follow-up (Figure 1H), the diameter and morphology of the left vertebral artery showed no significant changes. However, the hypoperfusion in the posterior circulation (Figure 1C, Red), which had been present before the procedure, largely returned to normal by the 8-month follow-up (Figure 1I, Green). Correspondingly, the patient's symptoms of left facial numbness and dysarthria resolved. At the 14-month clinical follow-up, the patient exhibited complete resolution of symptoms, with a modified Rankin Scale (mRS) score of 0, indicating an excellent functional outcome.

## Discussion

This case presents a 66-year-old male patient with a giant VADA in the left V4 segment, highlighting the diagnostic challenges, the role of advanced imaging techniques, and the efficacy of endovascular treatment using a flow diverter for patients with the CTA-negative VADA.

The pathophysiology of dissection is intricate and dynamic. The dynamic tearing of the vascular wall can lead to both ischemic and hemorrhagic events (1, 7, 8). As the dissected layer is in a constant state of destruction and repair, the morphology of the dissection can become complex and variable. Simultaneously, the intramural hematomas (9), both old and new, undergo continuous fusion, absorption, or disappearance. This dynamic evolution results in complex MRI presentations of the dissection, with the actual extent of the lesion potentially being underestimated (Figure 1A vs. Figure 1E) or even undetectable, as seen in cases of cryptogenic vascular dissections (5, 6). The presence of ischemic hypoperfusion in the posterior circulation, as demonstrated by the imaging findings, is a critical hemodynamic consequence of VAD. This hypoperfusion can lead to symptoms such as facial numbness and dysarthria, as experienced by the patient. Accurate assessment of the extent of dissection and the size of related VADA is crucial for guiding appropriate treatment and improving clinical outcomes.

Imaging plays a crucial role in the diagnosis and management of VADA. The results of CTA for VADA detection are influenced by multiple factors (10). Firstly, inadequate contrast dose, suboptimal injection rate, or individual differences in contrast metabolism may cause inadequate vessel enhancement, making it difficult to detect vascular changes. Secondly, if the scan is not performed during the optimal contrast phase, subtle vascular pathologies, such as dissections, may be missed. Thirdly, flow artifacts caused by high-velocity blood flow can lead to signal voids or blurred images, potentially masking small vascular abnormalities. Hence, CTA may miss some vascular dissections or dissection aneurysms (1) (Figure 1B). In contrast, DCE-CT permits dynamic observation of contrast agent distribution and clearance. This capability allows it to provide high-resolution imaging of anatomical structures and detailed tissue characterization, which in turn enables the detection of subtle pathological changes of VADA. Although MRI is highly sensitive for detecting dissection aneurysms (9), it also has limitations in accurately depicting the true morphology of the dissection or VADA. In this case, MRI detected a large lesion (Figure 1A), yet DCE-CT identified it as a giant VADA (Figure 1E). DCE-CT has higher accuracy in showing the full picture of the lesion and offers real-time guidance for treatment (Figures 1D–G). It is also superior to MRI in displaying the length of tortuous lesions, which is beneficial for selecting the appropriate stent size during surgery, as demonstrated in this case (Figure 1G).

In our previous report, we described a case where CTA was negative and MRI was equivocal, yet DSA revealed a significantly recurrent dissecting aneurysm (1). In contrast, the present case represents the first account of a patient with CTA-negative and MRI-equivocal findings, where even DSA failed to establish a definitive diagnosis. Ultimately, DCE-CT was instrumental in adequately demonstrating the VADA morphology and its proximal segment, playing a crucial role in formulating the treatment strategy. Thus, for patients presenting with CTA-negative results but with symptoms strongly suggestive of VADAs (1), VAD (10) or cryptogenic vascular dissection (5, 11), it remains imperative to perform enhanced DCE-CT for further evaluation, even if both CTA, MRI and DSA fail to disclose any evident abnormalities (5).

The treatment of dissection aneurysms and the prevention of recurrence are critical considerations (2, 12). In the context of dynamic vascular wall changes, the flow diverter helps redirect blood flow, stabilize the vessel wall, prevent further dissection, and mitigate the risk of aneurysm growth and rupture. This connection underscores how the flow diverter's mechanism synergizes with the pathological dynamics of dissection. For giant VADAs, treatment with multiple stents or flow diverter is recommended to minimize the risk of recurrence (1, 2, 12). Due to the limitations of imaging techniques, it is challenging to determine the exact origin of the dissection. Sometimes, even with flow diverter implantation, recurrence may still occur if the dissection origin is overlooked. Post-treatment CTA may show normal vascular morphology, yet recurrence can still happen (1). When patients present with new symptoms, recurrence of the lesion should be highly suspected. In such cases, MRI has a clear advantage over CTA in detecting recurrence, as well demonstrated in this study (1). One major reason is that MRI is more sensitive to new intramural hematomas.

In conclusion, this case highlights the significance of advanced imaging techniques such as DCE-CT in clinical practice. For doubted symptomatic patients, incorporating these technologies into routine practice can enhance VADA diagnostic accuracy, enable more precise device selection, and potentially improve patient outcomes. This underscores the need for further research to optimize their use in VADA management and refine treatment protocols.

## Limitations

DCE-CT has several limitations, including radiation exposure risk, susceptibility to motion artifacts, dependence on iodinated contrast agents with potential side effects, challenges in quantitative analysis, higher cost, and contraindications for certain patients.

## Data Availability

The datasets presented in this article are not readily available because of ethical and privacy restrictions. Requests to access the datasets should be directed to the corresponding author/s.
